# A novel long non-coding RNA SLNCR1 promotes proliferation, migration, and invasion of melanoma via transcriptionally regulating SOX5

**DOI:** 10.1038/s41420-024-01922-7

**Published:** 2024-04-01

**Authors:** Lele Cong, Qing Zhao, Hongyan Sun, Zilong Zhou, Yue Hu, Chunyi Li, Miao Hao, Xianling Cong

**Affiliations:** 1 Department of Dermatology, China-Japan Union Hospital of Jilin University, Changchun, Jilin China; 2 Department of Neurology, China-Japan Union Hospital of Jilin University, Changchun, Jilin China; 3 Department of Biobank, China-Japan Union Hospital of Jilin University, Changchun, Jilin China; 4 Institute of Antler Science and Product Technology, Changchun Sci-Tech University, Changchun, China; 5 Scientific Research Center, China-Japan Union Hospital of Jilin University, Changchun, Jilin China

**Keywords:** Melanoma, Prognostic markers

## Abstract

Steroid receptor RNA activator (SRA)-like non-coding RNA (SLNCR1) has been implicated in various tumorigenic processes, but the precise regulatory role in melanoma progression remains uncertain. We performed a comprehensive analysis to investigate the prognostic value of SLNCR1 expression in patients with melanoma by TCGA database and melanoma tissue samples *via* the Kaplan–Meier method. Subsequently, we conducted qRT-PCR and Fluorescence in Situ Hybridization (FISH) assays to identify SLNCR1 expression levels and localization in tissues and cells, respectively. Loss-of-function assays utilizing shRNAs vectors were used to investigate the potential impact of SLNCR1. Our data showed that SLNCR1 is significantly up-regulated in human malignant melanoma tissues and cell lines and functions as an oncogene. Silencing of SLNCR1 suppressed melanoma cell proliferation, migration, invasion, and inhibited tumorigenesis in a mouse xenograft model. Additionally, we employed bioinformatic predictive analysis, combined with dual-luciferase reporter analysis and functional rescue assays, to elucidate the mechanistic target of the SLNCR1/SOX5 axis in melanoma. Mechanistically, we discovered that SLNCR1 promotes EMT of human melanoma by targeting SOX5, as downregulation of SLNCR1 expression leads to a decrease in SOX5 protein levels and inhibits melanoma tumorigenesis. Our research offers promising insights for more precise diagnosis and treatment of human melanoma.

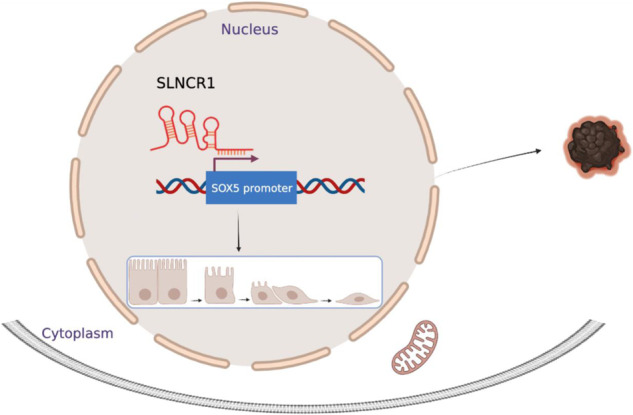

## Introduction

Melanocytes originate from the neural crest and migrate during embryonic development to various parts of the body, such as skin, uvea, meninges, and mucous membranes [1]. Their malignant transformation causes melanoma. Risk factors associated with melanoma include sun exposure [2], the number and size of melanocyte nevi [3], and a family history of the disease [4]. Melanoma is the most lethal form of skin cancer, and early detection is key to successful treatment [5, 6]. Approximately 75% of melanomas are highly metastatic, and the incidence has increased annually in recent years [7]. Therefore, the development of early diagnostic and prognostic markers for melanoma is important for improving human health.

Long non-coding RNA (lncRNA), a class of non-protein-coding transcripts, are longer than 200 nts [8]. Growing evidence revealed that lncRNAs function as oncogenes or tumor suppressors, which can participate in tumor cell proliferation, migration, invasion, and apoptosis, through transcriptional and post-transcriptional regulatory mechanisms [9, 10].

Steroid receptor RNA activator (SRA)-like non-coding RNA (SLNCR1) is a recently identified lncRNA located on human chromosome 17q24.3 [11]. Despite being a relatively novel lncRNA, several studies have suggested that SLNCR1 expression is upregulated in various types of cancers, such as non-small cell lung cancer [12], breast cancer [13], cervical cancer [14], and papillary thyroid carcinoma [15], and may contribute to tumor progression. In a recent study, SLNCR1 was found to be involved in regulating melanoma invasion, but its specific functions and underlying mechanisms in melanoma remain unclear and merit further investigation [16].

This study focuses on exploring the role and mechanism of SLNCR1 and its downstream target gene in regulating the development of malignant melanoma. The transcription factor SRY-related high-mobility-group box5 (SOX5), a member of the SOX family, plays an important role in the regulation of gene transcription. SOX5 is essential for embryonic development and differentiation [17], and recent studies have highlighted its involvement in regulating proliferation, invasion, migration, metastasis, and epithelial-to-mesenchymal transition (EMT) in a variety of cancers [18–21]. Moreover, SOX5 has been shown to be associated with regulation by microphthalmia transcription factor (MITF) in melanoma [22].

In this study, we used the public database TCGA and melanoma tissue samples to clarify the expression and prognostic value of SLNCR1. Additionally, we assessed the functions of SLNCR1 both in vitro and in vivo using cell lines and mouse models. Finally, we explored the potential regulatory mechanisms by which SLNCR1 is involved in the development of melanoma progression. Our findings are expected to contribute new strategies for early diagnosis and precision treatment of melanoma.

## Results

### SLNCR1 is overexpressed and has a poor prognosis in malignant melanoma tissues and cell lines

**Fig. 1:**
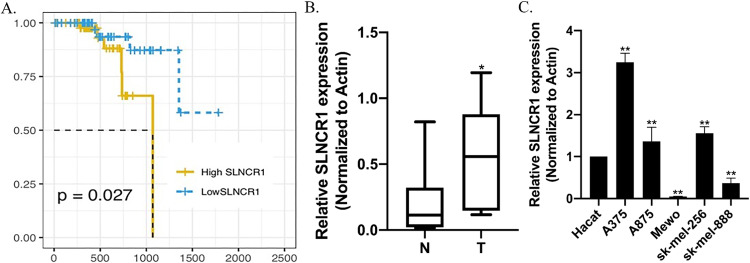
SLNCR1 was upregulated in melanoma tissues and cell lines. **A** Kaplan–Meier OS curves of SLNCR1 expression from TCGA database. **B** SLNCR1 expression was determined using qPCR in 27 paired melanoma tissues and adjacent normal tissues. **C** Expression of SLNCR1 was assessed using qPCR in melanoma cell lines and normal cell lines. *p < 0.05; **p < 0.05; ***p < 0.005; N Normal, T Tumor.

To investigate the relationship between SLNCR1 expression and overall survival (OS), Kaplan–Meier analysis was performed using the TCGA database. Melanoma tissues were stratified into low or high groups based on the median expression of SLNCR1. Patients with high expression had worse OS than the low expression group (*p* = 0.027) (Fig. 1A). Additionally, 27 pairs of melanoma and adjacent tissues were collected and performed by qRT-PCR. SLNCR1 expression was significantly upregulated in melanoma tissues compared to adjacent tissue (Fig. 1B). This finding was further validated using various melanoma cell lines, including A375, A875, Mewo, Sk-mel-256, Sk-mel-888, and human immortalized keratinocytes (Hacat). The results demonstrated that the SLNCR1 expression was significantly increased in A375, A875, and Sk-mel-256 cell lines compared to Hacat cells (Fig. 1C). We have collected clinicopathology characteristics from 27 malignant melanoma patients in our hospital, the correlation between SLNCR1 expression and clinicopathologic characteristics of melanoma patients was also analyzed. The results showed that patients with high SLNCR1 expression may have a higher risk of P53 mutation (*p* = 0.0335) and lymph node metastasis (*p* = 0.0377) than patients with low expression (Table 1). SLNCR1 may act as an oncogene and be involved in melanoma progression.

**Table 1:** Correlation between SLNCR1 expression level and patients’ clinicopathological features of melanoma (*P < 0.05).

SLNCR1 expression			
Clinical parameter	High expression cases (*n* = 14)	Low expression cases (*n* = 13)	Chi-squared test *P*-value
Age (years)			0.7064
≤63	8	6	
>63	6	7	
Gender			>0.9999
Male	6	6	
Female	8	7	
Tumor size			0.5825
≥5 cm	8	5	
<5 cm	5	6	
Missing	1	2	
TNM stages			0.3688
I/II	1	0	
III/IV	0	1	
Missing	13	12	
P53			0.0335*
Yes	3	1	
No	5	11	
Missing	6	1	
Ras			0.5113
Yes	1	0	
No	11	12	
Missing	2	1	
Lymphatic metastasis			0.0377*
Yes	7	1	
No	1	4	
Missing	6	8	
Ki67			0.5680
Yes	13	11	
No	0	1	
Missing	1	1	
Vascular invasion			0.0734
Yes	4	0	
No	5	4	
Missing	5	9	
Perineural invasion			0.2268
Yes	2	0	
No	6	4	
Missing	6	9	

### SLNCR1 promotes melanoma cell proliferation, migration, invasion, and EMT

**Fig. 2:**
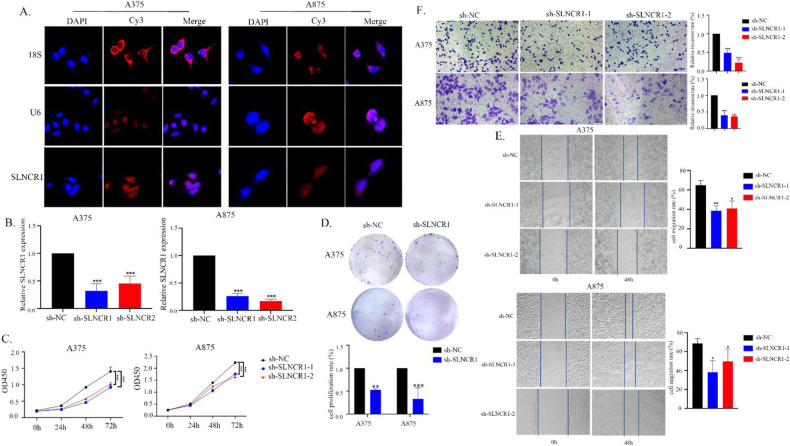
SLNCR1 promotes proliferation, migration, and invasion. **A** The FISH assay revealed the distribution of SLNCR1 in A375 and A875 cell lines. **B** Expression of SLNCR1 in the sh-SLNCR-transfected A375 and A875 cells was determined via qPCR. **C**, **D** CCK-8 and colony formation assays were conducted to determine the cell proliferation rate upon SLNCR1 silencing. **E** The effect of SLNCR1 interference on cell migration was estimated via wound healing assay. **F** Invasiveness of sh-SLNCR1- transfected A375 and A875 cell lines was evaluated via Transwell assay.

To identify how SLNCR1 expression regulates melanoma progression, we first localized SLNCR1 in the studied cell lines using RNA FISH assay. The results suggested that SLNCR1 was mainly located in the nucleus and a small amount distributed in the cytoplasm, indicating that SLNCR1 may promote melanoma progression at the level of transcriptional regulation (Fig. 2A). To determine the function of SLNCR1 in melanoma, loss-of-function assays were performed in A375 and A875 cells, respectively. As shown in Fig. 2B–D, SLNCR1 expression was significantly decreased after sh-SLNCR1 transfection which inhibited melanoma cell proliferation and colony formation. Additionally, the regulatory effect of SLNCR1 on cell metastasis was examined *via* wound healing and Transwell assays. Compared with the control group, the migration and invasion abilities were significantly reduced after SLNCR1 inhibition (Fig. 2E, F). Taken together, these results suggest that high SLNCR1 expression promotes melanoma proliferation, migration, and invasion.

**Fig. 3:**
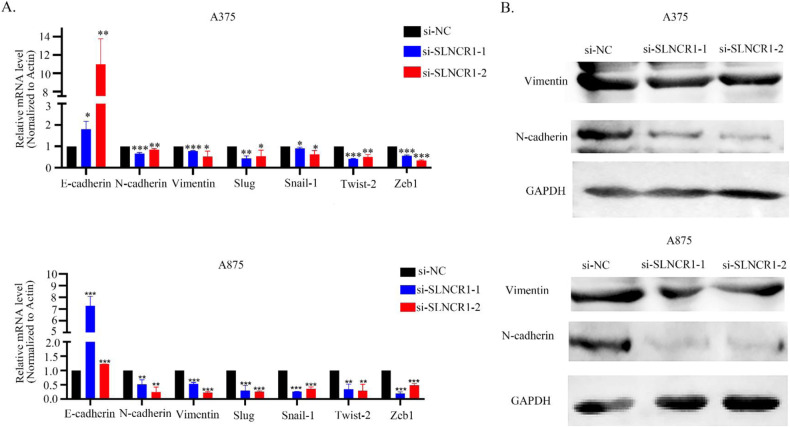
SLNCR1 promotes EMT. **A** Expression levels of EMT markers in the SLNCR1 knocked down A375 and A875 cell lines were examined via qPCR. **B** Expression levels of EMT marker in the SLNCR1 knocked down A375 and A875 cells were examined via western blot analysis.

Epithelial-mesenchymal transition (EMT) with cell invasion, we investigated the changes in EMT markers after SLNCR1 knockdown to determine whether SLNCR1 was related to melanoma EMT. With SLNCR1 inhibition, both mRNA and protein levels of epithelial markers increased, while those of mesenchymal markers decreased, suggesting that SLNCR1 may promote EMT in melanoma (Fig. 3A, B).

### SOX5 is the downstream target molecule of SLNCR1

**Fig. 4:**
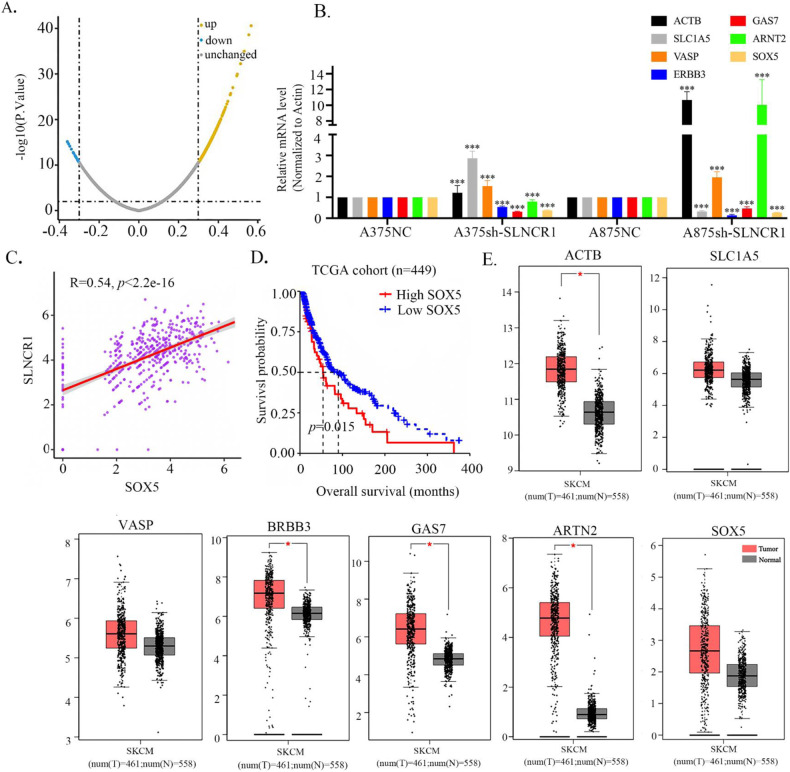
Search for the downstream target molecule of SLNCR1. **A** Volcano plot showed the differentially expressed transcriptional factors after SLNCR1 was knocked down. **B** Expression levels of the positively and negatively regulated molecules were detected after SLNCR1 was knocked down via qPCR. **C** Correlation between SLNCR1 and SOX5 expression level. **D** Kaplan–Meier overall survival of SOX5 in melanoma from TCGA database. **E** Expression levels of the genes in melanoma tissue predicted by GEPIA.

To investigate differentially expressed genes associated with SLNCR1 in melanoma progression, a |logFC| > 0.3 and *p* < 0.05 were considered the threshold for the SLNCR1-related genes by TCGA data using the R package (version 3.6.1). A total of 9889 upregulated and 3457 downregulated genes were screened (Fig. 4A). The top 20 differentially regulated candidate genes are summarized in Table 2.

**Table 2:** The top 20 genes that positively or negatively correlated with SLNCR1 selected from the TCGA database.

Positively correlated			Negatively correlated		
Gene	Correlation	*P* value	Gene	Correlation	*P* value
ERBB3	0.5658	2.6244E−41	PACS1	−0.3596	7.4087E−16
ABTB2	0.5539	2.6190E−39	MOB3A	−0.3594	7.6295E−16
GAS7	0.5308	1.1442E−35	RHOG	−0.3539	2.2358E−15
PLP1	0.5246	9.5499E−35	MICAL2	−0.3477	7.3384E−15
ARNT2	0.5124	5.8546E−33	ACTB	−0.3397	3.2492E−14
EXTL1	0.4960	1.0968E−30	SLC1A5	−0.3305	1.7037E−13
TRIM2	0.4934	2.4585E−30	TNFAIP8L1	−0.3280	2.6229E−13
POLR2F	0.4732	1.0261E−27	GMIP	−0.3214	8.3210E−13
SHC4	0.4628	1.9512E−26	VASP	−0.3183	1.4113E−12
SOX5	0.4606	3.6523E−26	TBC1D2	−0.3141	2.8410E−12

To validate the results of the analysis, we selected seven differentially expressed genes (four upregulated and three downregulated) and measured their expression levels in the SLNCR1-downregulated cell lines *via* qRT-PCR. As shown in Fig. 4B, among the positively correlated genes in both A375 and A875 cells, SOX5 expression was most significantly decreased after SLNCR1 knockdown. Based on TCGA data, we found that SLNCR1 expression was positively associated with SOX5 expression in melanoma (R = 0.54, *p* < 0.01) (Fig. 4C). Meanwhile, we noticed that high SOX5 expression was associated with shorter OS (*p* = 0.015) (Fig. 4D). Furthermore, results of GEPIA analysis showed that ACTB, SLC1A5, VASP, BRBB3, GAS7, ARTN2, and SOX5 were significantly increased in melanoma tissues (*p* < 0.05) (Fig. 4E). Therefore, SOX5 was selected as a candidate downstream target molecule of SLNCR1.

### SOX5 is highly expressed in melanoma and regulates EMT

We next investigate the relationship between SOX5 expression and clinicopathologic features of patients. As shown in Table 3, SOX5 expression was significantly associated with N stage (*p* = 0.016), age (*p* = 0.046), tumor tissue site (*p* = 0.005), Breslow depth (*p* = 0.021), DSS event (*p* = 0.04), and PFI event (*p* = 0.004), indicating that SOX5 may participate in the occurrence and development of melanoma.

**Table 3:** Correlation between SOX5 expression and melanoma patients’ clinicopathological features based on the TCGA dataset (*P < 0.05, **P < 0.005).

Characteristic	Low expression of SOX5 (*n* = 235)	High expression of SOX5 (*n* = 236)	*P*
T stage, *n* (%)			0.295
T1	21 (5.8%)	20 (5.5%)	
T2	34 (9.3%)	45 (12.4%)	
T3	43 (11.8%)	48 (13.2%)	
T4	85 (23.4%)	68 (18.7%)	
N stage, *n* (%)			0.016*
N0	113 (27.3%)	122 (29.5%)	
N1	30 (7.2%)	44 (10.6%)	
N2	26 (6.3%)	23 (5.6%)	
N3	38 (9.2%)	18 (4.3%)	
M stage, *n* (%)			0.954
M0	206 (46.5%)	212 (47.9%)	
M1	13 (2.9%)	12 (2.7%)	
Age, *n* (%)			0.046*
≤60	114 (24.6%)	138 (29.8%)	
>60	116 (25.1%)	95 (20.5%)	
Tumor tissue site, *n* (%)			0.005*
Extremities	89 (21.2%)	108 (25.8%)	
Trunk	80 (19.1%)	91 (21.7%)	
Head and Neck	27 (6.4%)	11 (2.6%)	
Other Specify	10 (2.4%)	3 (0.7%)	
Breslow depth, *n* (%)			0.021*
≤3	79 (21.9%)	106 (29.4%)	
>3	97 (26.9%)	78 (21.7%)	
OS event, *n* (%)			0.051
Alive	135 (29.1%)	112 (24.1%)	
Dead	98 (21.1%)	119 (25.6%)	
DSS event, *n* (%)			0.04*
Alive	146 (31.9%)	121 (26.4%)	
Dead	85 (18.6%)	106 (23.1%)	
PFI event, *n* (%)			0.004**
Alive	92 (19.8%)	61 (13.1%)	
Dead	141 (30.4%)	170 (36.6%)	

**Fig. 5:**
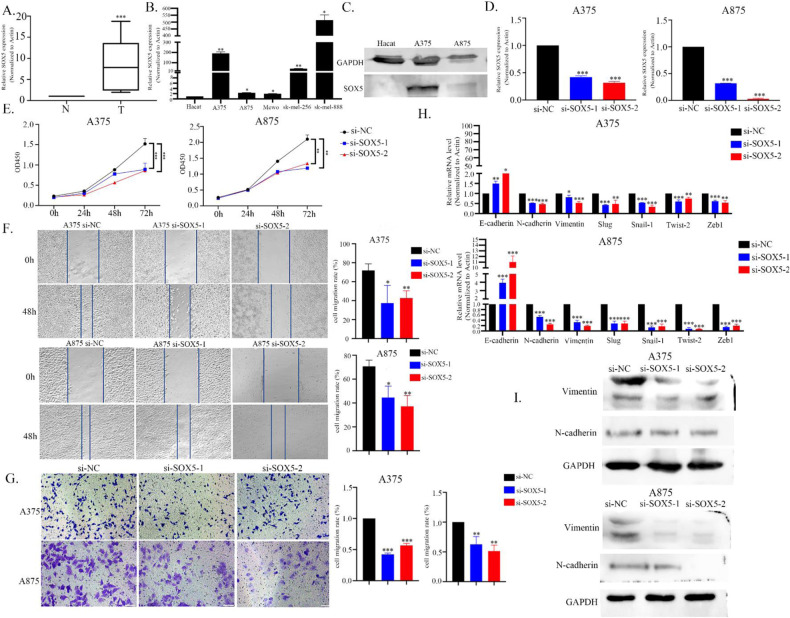
SOX5 expression was upregulated in melanoma tissues and cell lines. **A** SOX5 expression levels were determined via qPCR in the 27 paired melanoma tissues and adjacent normal tissues. **B**, **C** Both mRNA and protein expressions of SOX5 were measured via qPCR in the melanoma cell lines and normal cell lines. **D** Effects of SOX5 knockdown was determined using qPCR in A375 and A875 cell lines. **E** Effects of SOX5 inhibition on cell proliferation using CCK8 assay. Migration (**F**) and invasion (**G**) in A375 and A875 cells after SOX5 inhibition were observed by wound healing and transwell assays. mRNA expression (**H**) and protein expressions of EMT markers (**I**) in A375 and A875 cell lines after SOX5 inhibition were observed by qPCR and western blot assay.

To further investigate the role of SOX5 in melanoma, we measured SOX5 expression levels in the 27 pairs of melanoma tissues and cells. As shown in Fig. 5A, SOX5 expression was significantly increased in melanoma tissues compared to normal tissues. Similar results were found in the cell lines, where both mRNA and protein expression of SOX5 were greatly increased in A375 and A875 cells (Fig. 5B, C). To determine the biological function of SOX5, siRNAs were transfected into these two melanoma cell lines (Fig. 5D). We found a significantly inhibitory effect on proliferation in the SOX5 silencing group compared to the control (Fig. 5E).

Furthermore, to investigate migration and invasion in melanoma cells, wound healing and transwell assays were performed. As shown in Fig. 5F, G, compared to the control group, SOX5 inhibition significantly suppressed cell migration in both A375 and A875 cell lines. To further clarify the regulatory role of SOX5 on EMT, qPCR and western blot assays were performed. As shown in Fig. 5H, I, SOX5 inhibition resulted in a significant increase of E-cadherin, and a decrease in mesenchymal markers, including N-cadherin and vimentin. These results indicate that SOX5 may function as an oncogene involved in melanoma development.

### SOX5 over-expression reverses si-SLNCR1 induced proliferation, migration, and EMT in vitro

**Fig. 6:**
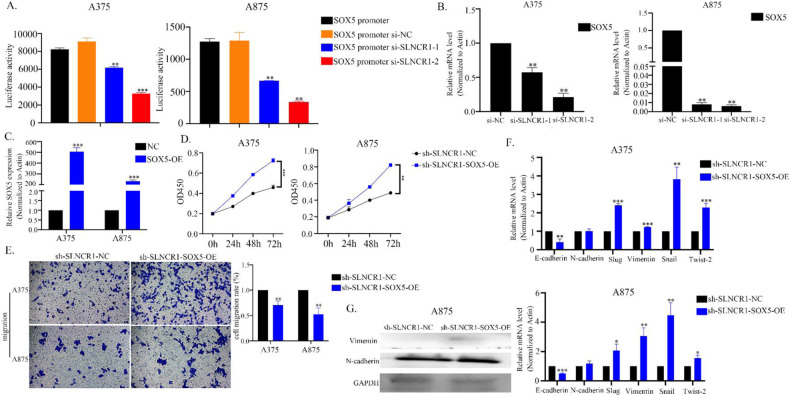
SOX5 over-expression reverses si-SLNCR1 induced proliferation, migration, and EMT in vitro. **A** SLNCR1 regulates the transcriptional activity at the SOX5 promoter region. **B** SOX5 expression was detected via qPCR after SLNCR1 was knocked down. **C** Overexpression of SOX5 was detected using qPCR. **D** CCK8 assay showed the proliferation of A375 and A875 cell lines when SOX5 was overexpressed. **E** Migration rates of A375 and A875 cells after SOX5 overexpressed. mRNA (**F**) and protein (**G**) expression of EMT markers after SOX5 overexpressed were observed by qPCR and western blot assays.

To investigate the potential interactions between SLNCR1 and SOX5, a dual-Luciferase reporter assay was performed. The results showed that silencing of SLNCR1 inhibited the transcriptional activity of the SOX5 promoter (Fig. 6A). Furthermore, SOX5 expression was significantly decreased after SLNCR1 knockdown in melanoma cells (Fig. 6B), indicating that SOX5 may be a downstream target molecule of SLNCR1. We then overexpressed SOX5 in SLNCR1-silenced melanoma cells to determine its effects on cell proliferation, migration, and EMT markers (Fig. 6C). As shown in Fig. 6D, E, SOX5 overexpression reversed the inhibition of cell proliferation and migration induced by SLNCR1 silencing. In addition, SOX5 overexpression had the opposite effect on the changes in EMT markers induced by SLNCR1 silencing (Fig. 6F, G). These findings suggest that SLNCR1 may regulate melanoma progression by targeting SOX5.

### SLNCR1 down-regulation suppresses melanoma growth in vivo

**Fig. 7:**
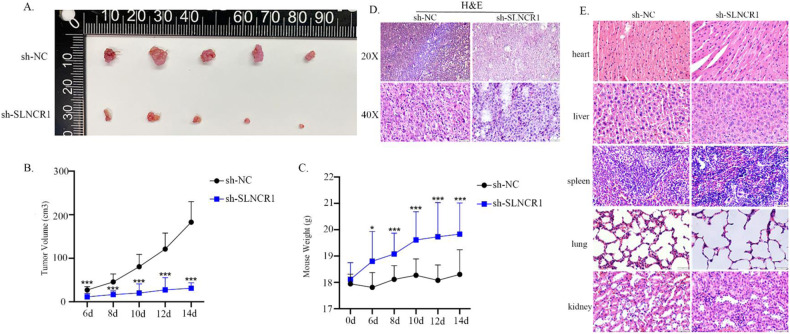
SLNCR1 involved in melanoma progression in vivo. **A** Photographs of tumors collected from different groups. **B**, **C** Tumor volume and mouse weight were measured. **D** H&E staining of the primary tumors. **E** H&E staining of mice internal organ tissues. Original magnifications 40×.

To investigate the anti-tumor effects of SLNCR1 inhibition, we inoculated Balb/c mice with A375 cells stably expressing lentiviral sh-SLNCR1 to suppress SLNCR1 expression. Mice were randomly divided into two groups, one bearing sh-SLNCR1 cells and the other bearing sh-NC cells. After the treatment, the body weights and tumors were measured. As shown in Fig. 7A, compared to the normal group, significant tumor inhibitory effects were observed in the SLNCR1 inhibition group. After 2 weeks, the SLNCR1 inhibition group showed significantly decreased volumes and weights compared to the control (Fig. 7B, C). Enlargement and mitotic figures of nuclei were observed using H&E staining, indicating that subcutaneous tissue was tumor tissue (Fig. 7D). We also examined the heart, liver, spleen, lungs, and kidneys using histology. As shown in Fig. 7E, the knockdown of SLNCR1 had no obvious effect on the tissues of the mice internal organs. These results suggest that SLNCR1 could serve as a potential candidate target for melanoma treatment.

### SLNCR1/SOX5 promotes melanoma by regulating EMT mechanism

**Fig. 8:**
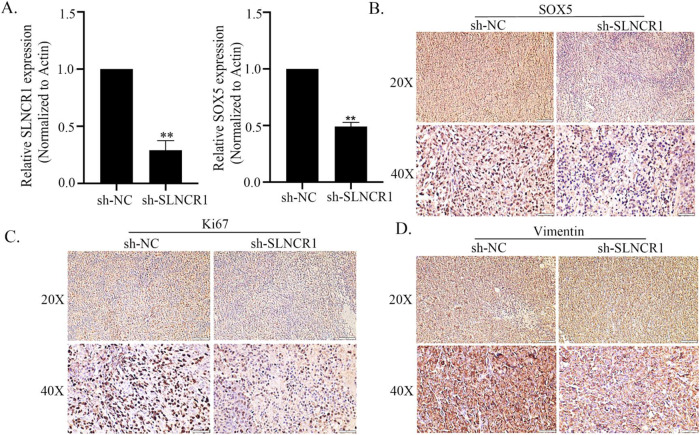
SLNCR1/SOX5 promotes melanoma by regulating EMT mechanism. **A** Expressions of SLNCR1 and SOX5 in the subcutaneous tumor tissue by qPCR. Expression of SOX5 (**B**), Ki67 (**C**), and Vimentin (**D**) in the subcutaneous tumor tissue by IHC staining.

In this study, we employed qPCR to investigate the in vivo relationship between SLNCR1 and SOX5. Our results revealed that SOX5 expression was significantly reduced in the sh-SLNCR1 group compared to the control group, indicating that SLNCR1 inhibition led to the down-regulation of SOX5 (Fig. 8A). To further validate this finding, we conducted an immunohistochemical (IHC) analysis to measure the protein expression of SOX5. The results were consistent with the qPCR findings, indicating that SLNCR1 inhibition resulted in decreased SOX5 expression (Fig. 8B). We also evaluated the expression of two epithelial-mesenchymal transitions (EMT) markers, Ki67 and Vimentin. Our findings showed that the knockdown of SLNCR1 resulted in the suppression of Ki67 and Vimentin expressions (Fig. 8C, D), suggesting that SLNCR1/SOX5 axis may promote melanoma growth in vivo *via* the regulation of the EMT pathway.

## Discussion

Melanoma is a highly metastatic skin cancer [23]. Understanding the molecular pathways that govern the invasion of nevus melanocytes and the transformation of these cells into melanoma is crucial for fully elucidating the mechanisms underlying melanoma genesis. Many studies have shown that lncRNAs regulate various cellular processes, including cell proliferation, differentiation, migration, and invasion. Several lncRNAs, such as HOTAIR, MALAT1, and BANCR have been reported to be dysregulated in melanoma [24].

SLNCR1 is a newly discovered lncRNA that may play an essential role in the occurrence and development of melanoma. For example, SLNCR1 has been shown to promote melanoma invasion and growth through its interaction with androgen receptor and EGR1 [16, 25]. In our study, we further observed the function and role of SLNCR1 in malignant melanoma. Our findings revealed that SLNCR1 was overexpressed in melanoma tissues and cells, and analysis of TCGA database showed that high SLNCR1 expression was associated with poor overall survival rates. In this study, compared to Hacat cells, SLNCR1 is highly expressed in most of malignant melanoma cell lines, such as A375, A875, and sk-mel-256 cells. While Mewo and Sk- mel-888 cell lines obtained opposite results (Fig. 1C). This may be due to the different genetic backgrounds of different cell lines, including donor origin, mutation sites, and other species specificities. These results suggest that we could classify malignant melanoma into different subtypes based on SLNCR1 expression for precision diagnosis in the future.

Furthermore, knockdown of SLNCR1 inhibited the proliferation, migration, and invasion of melanoma cells in vitro. Since cell invasion is closely related to EMT [26], we evaluated EMT markers following SLNCR1 knockdown. We found that the expression of epithelial marker E-cadherin was up-regulated, while the mesenchymal markers N-cadherin and vimentin were down-regulated, suggesting that SLNCR1 knockdown suppressed the occurrence of EMT.

Different localization of lncRNA in cells determines how they participate in tumor progression [27]. The lncRNA in the nucleus usually participates in melanoma progression in the transcriptional regulation way by modulating their downstream transcription factors [28]. It was found that lncRNA BASP1-AS1 can interact with YBX1, activating the Notch signaling pathway and driving migration in melanoma [29]. The LncRNA in the cytoplasm is involved in cancer in the post-transcriptional regulation way by interacting with specific proteins and RNAs [30]. LncRNA HOXD-AS1 can bind to EZH2 and inhibit RUNX3 expression through epigenetic regulation [31]. Furthermore, there is considerable evidence that competition for miRNAs plays an important part in LncRNA regulation. For example, lncRNA MALAT1 could bind to miR-23a, promoting proliferation, migration, and invasion of melanoma [32]. Although diverse functions have been characterized, specific insights relating to the lncRNAs in tumorigenesis are poorly understood.

SLNCR1 was mainly localized in the nucleus of cells using RNA FISH. Therefore, we inferred that SLNCR1 promotes melanoma genesis through transcriptional regulation. To identify the downstream transcription factors of SLNCR1, we searched the TCGA database and found that SOX5 may be a target molecule of SLNCR1. SOX5 plays an essential role in cell-fate decision and differentiation. For example, SOX5 could inhibit glioma proliferation in vitro, while SOX5 knockdown elevates the ability of glioma growth in mouse models [33]. Moreover, SOX5 has been identified as a predictor of poor prognosis in lung adenocarcinoma, and SOX5 is known to promote lung adenocarcinoma progression and metastasis through EMT [34].

In our study, GEPIA predicted that SOX5 was overexpressed; therefore, qPCR and western blot analyses were performed. SOX5 was highly expressed in melanoma tissues and cell lines compared to controls. Furthermore, SOX5 silencing suppressed the proliferation and migration capability of melanoma cells, but not invasion. We found that the expression of SOX5 decreased after SLNCR1 knockdown, and dual-luciferase reporter assays showed that SLNCR1 silencing reduced the transcriptional activity of the SOX5 promoter region, which verified the regulatory relationship between SLNCR1 and SOX5. Overexpression of SOX5 reversed the inhibition of proliferation and migration of melanoma cells induced by SLNCR1 silencing.

SLNCR1, a novel lncRNA with potential cancer-promoting function, which downstream regulated target genes and functions are still unclear. In our study, we found downstream target SOX5 of SLNCR1 from the perspective of lncRNA transcriptional regulation and clarified that SLNCR1/SOX5 axis promotes invasion and metastasis of melanoma through EMT, suggesting that SLNCR1 could be used as a potential biomarker and therapeutic target for melanoma.

This study successfully constructed a subcutaneous tumorigenesis model in nude mice using SLNCR1-stable knockdown A375 cells. Our results showed that SLNCR1 silencing inhibited melanoma proliferation and EMT in vitro. Furthermore, SLNCR1 inhibition decreased both mRNA and protein expression of SOX5, which were consistent with the previous results.

In conclusion, our study revealed that SLNCR1 promotes EMT of melanoma by targeting SOX5, these findings suggest that SLNCR1 may serve as a potential marker for assessing the incidence and prognosis of melanoma.

## Materials and methods

### Data collection and prognostic model

RNA-seq transcriptomic data and clinical data for 472 melanoma patients were downloaded from the Cancer Genome Atlas (TCGA) database (https://portal.gdc.caner.gov). Of these patients, 449 had complete survival data, and we classified them into high- and low-risk groups based on their risk scores, with the median value serving as the threshold. Kaplan–Meier method was performed for overall survival (OS).

We retrieved gene expression data for skin malignant melanoma from the official TCGA database using GDC-client and obtained the original RNA expression data. To standardize the data, we converted it to TPM format, resulting in a total of 17,580 genes, including SLNCR1, after filtering out those with very low expression values. We then performed log2 transformation on these genes and conducted a correlation analysis to investigate the relationship between SLNCR1 and other coding genes.

### GEPIA analysis

GEPIA (http://gepia.cancer-pku.cn/index.html) is an open-access database that enables in-depth analysis of gene expression data from TCGA. GEPIA was performed to investigate candidate genes associated with SLNCR1.

### Patients and samples

A total of 27 pairs of melanoma and adjacent tissues were collected from China-Japan Union Hospital of Jilin University (2009–2021). These patients were diagnosed with melanoma through pathological examination and had not undergone any chemotherapy or radiotherapy before surgical resection. The adjacent tissues were defined as having no tumor at the surgical margin. Table 1 provides a summary of the clinicopathological characteristics of the patients included in this study. Ethical approval was obtained by the China-Japan Union Hospital of Jilin University.

### Cell culture

The human malignant melanoma cell lines A375 and A875 were purchased from ATCC, while the Hacat, Mewo, Sk-mel-256, and Sk-mel-888 cell lines were maintained in our laboratory. All cell lines were cultured in DMEM with 10% fetal bovine serum.

### Silencing of target gene expression by siRNA and shRNA

Small interfering RNA (siRNA) targeting SOX5 and control siRNA were designed by Genepharma (Shanghai, China). According to the manufacturer’s instructions, cell transfections were performed using Lipofectamine 2000 (Invitrogen, USA). qPCR analysis to assess the efficacy of knockdown. We purchased the SLNCR1 lentiviral vector and empty lentiviral vector from Hanheng (Shanghai, China), infected cells and selected with 1 μg/ml puromycin (Cat#P8230, Solarbio, China) to obtain stable knockdown cell lines.

### RNA extraction and real-time quantitative PCR (qPCR)

Total RNA was extracted using Trizol Reagent (Invitrogen) following the manufacturer’s instructions. cDNA was reverse transcripted using an RR047A kit (Takara, Japan). Real-time PCR was performed with SYBR Green (Roche, USA) on a 7500 Fast Real-Time PCR system (ABI, USA). Gene expression was quantified using the 2^−ΔΔCt^ method, with actin expression used as the internal control for normalization. The primers used are listed in Table 4.

**Table 4:** Primer list.

Primer	Sequence (5’-3’)
SLNCR1	F: GGACCCCTTAACGTGGATTAC
	R: AAATACCTCCAGCTTGGCG
SOX5	F: TGCCTGGTGGATGGCAAAAAGC
	R: TGCTAGACACGCTTGAGTGC
si-SLNCR1	si-NC: UUC UCC GAA CGU GUC ACG UTT
	si-SLNCR1-1: GGA UAC AGA GUG AAU AGU UTT
	si-SLNCR1-2: CCU UGG AAU AGU AAC UCU UTT
si-SOX5	si-NC: UUC UCC GAA CGU GUC ACG UTT
	si-SOX5-1: GCC AUU AAU GAU UCC CGU ATT
	si-SOX5-2: GCC AUA UUA UGA GGA GCA ATT
Actin	F: GTTGCTATCCAGGCTGTGCT
	R: AGCACTGTGTTGGCGTACAG

### Cell counting kit-8 (CCK8) assay

To assess the effect of SLNCR1 on cell proliferation, cell Counting Kit-8 (CCK-8) assay was performed. Cells were seeded in 96-well plates at a density of 2 × 10^3 cells/well. CCK-8 solution was added and incubated for 2 h. The absorbance was observed by a microplate at 450 nm (Bio-tek).

### Wound healing assay

To evaluate the effect of SLNCR1 on melanoma cell migration, a wound healing assay was performed Approximately 5 × 10^4 cells were seeded in a 6-well plate. After 48 h, the cells were wounded to create a straight scratch. The wells were then washed and treated with fresh medium containing 2% FBS. Digital microscopy (Olympus) was used to capture images of each wound at 0, 24, and 48 h. The wound space was calculated by subtracting the scratch widths at 24 or 48 h from the width at 0 h using the MRI tool in Image J.

### Transwell invasion assay

To determine the invasive abilities, Transwell invasion assay was performed. A total of 5 × 10^3 cells without serum were added to the upper chamber of Transwell inserts (Corning) coated with matrigel (BD Biosciences). DMEM medium containing 10% FBS was added to the lower chamber as a chemoattractant. After 48 h, the invaded or migrated cells were fixed and stained. Non-invading cells were removed. The invasive abilities were evaluated in five randomly selected fields.

### Western blot assay

The cells were harvested and lysed, measured using bicinchoninic acid (BCA) protein assay kits (Beyotime). Then, the samples were subjected to SDS-PAGE and transferred. The blot was incubated with primary antibodies against E-cadherin (1:1000, cat#198751, Sangon), N-cadherin (1:1000, cat#D199282, Sangon), vimentin (1:1000, cat#5741, CST), Slug (1:1000, cat#9585, CST), SOX5 (1:1000, cat#13216-1-AP, Proteintech), and GAPDH. The bands were visualized using a chemiluminescence imaging system (SAGE).

### Luciferase reporter assay

In the initial step, a dual-luciferase reporter plasmid, pGL3-basic, containing the promoter region (1-600) of SOX5 was synthesized by Comate Biosciences (pGL3- basic-SOX5-promoter). Cells were seeded at 5 × 10^4 cells/well in 24-well plates and allowed to grow overnight. Then, the cells were co-transfected with pGL3-Basic- SOX5-promoter, SLNCR1-siRNA, and Renilla plasmids (Comate Biosciences, Shanghai, China). After transfection, the cells were lysed and the supernatant was collected. Firefly luciferase reagent was added, and the luciferase activity was measured using a Multilabel Reader (PerkinElmer) and normalized to the renilla luciferase activity.

### Fluorescence in situ hybridization (FISH)

The FISH probe was synthesized by Genepharma. According to the manufacturer’s instructions, cells were seeded onto confocal plates and incubated overnight. Then, the cells were washed with PBS and fixed. The cells were then treated with protease K, glycine, and acetylation reagent, incubated with hybridization solution, and probe-labeled SLNCR1. The cover glass was washed with PBST and the nucleus was stained with DAPI (1:800) diluted in PBST. The confocal plates were washed, sealed, and observed by fluorescence microscope (Olympus).

### Hematoxylin-eosin (H&E) staining

The organs were fixed overnight. Subsequently, the tissues were stained with hematoxylin and eosin (H&E) and observed by microscope (Olympus, Tokyo, Japan).

### Immunohistochemistry (IHC)

Immunohistochemical (IHC) staining was performed on dissected subcutaneous tumors from mice. The slides were deparaffinized and dehydrated with xylene and graded alcohols, respectively. Antigen retrieval was performed, and the slides were incubated with primary antibodies against SOX5, vimentin, and Ki67, followed by incubation with a secondary antibody (DAKO) and visualization using DAB solution.

### Xenograft model assay

A375 melanoma cells stably transduced with either sh-NC (control) or sh-SLNCR1 were cultured in complete media supplemented with 1 μg/ml puromycin until reaching confluence. Ten 6-week-old female BALB/c nude mice were subcutaneously injected with A375-NC or A375-sh-SLNCR1 transduced cells at a dose of 1 × 10^7/100 μL into the right back. Tumor size was measured on days 6, 8, 10, 12, and 14 after injection, and the animals were euthanized when the tumor volumes reached about 100 mm^3^. Tumor volume was calculated using the formula V(mm^3^) = (L × W2) × 0.5 (L: tumor length, W: width). All the animal experiment was approved by Institional Animal Ethics Committee.

### Statistic analysis

Statistical analysis was performed using GraphPad Prism 8.0 software (GraphPad Software, Inc.). Student’s *t* test was used to compare the means of two independent samples. The associations between SLNCR1 mRNA levels and clinical pathological parameters of melanoma patients were assessed using the Chi-square (χ^2^) test. Survival curve analyses were performed using the R package (version 3.6.1). The expression of SOX5 was estimated using the GEPIA and TCGA databases. Statistical significance was defined as follows: *p < 0.05; **p < 0.005; and ***p < 0.0005.

### Supplementary information

Original Data File

## Data Availability

All data generated or analyzed during this study are included in this published article.
