# Supplementary material for: A novel long non-coding RNA SLNCR1 promotes proliferation, migration, and invasion of melanoma via transcriptionally regulating SOX5
Source: Cell Death Discov. 2024 Apr 1;10:160. doi: 10.1038/s41420-024-01922-7 (PMC10984963; doi:10.1038/s41420-024-01922-7)
Supplement: Supplementary file 1 — Original Data File [file 41420_2024_1922_MOESM1_ESM.pdf]

Figure 3

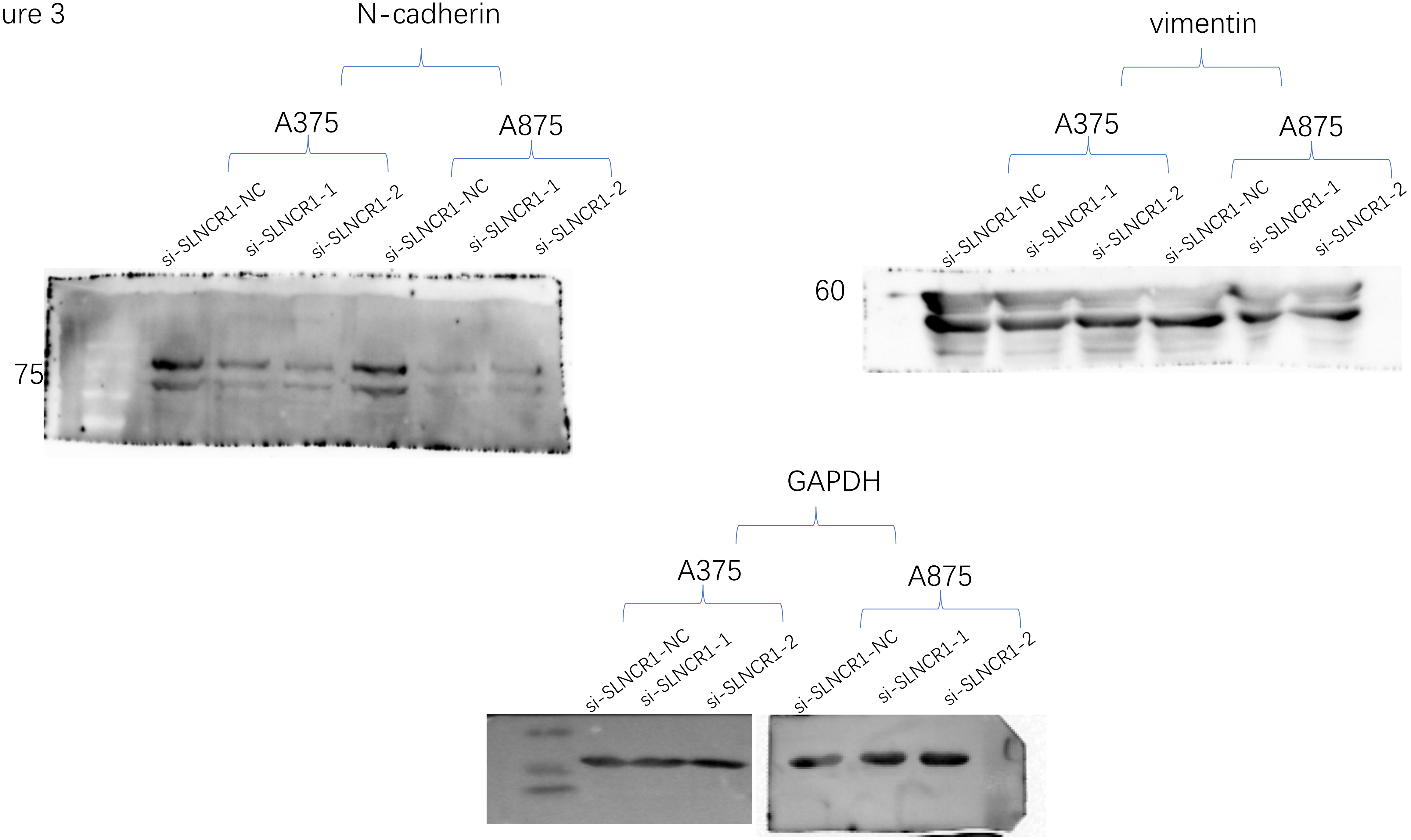

N-cadherin
A375
A875
si-SLNCR1-NC
si-SLNCR1-1
si-SLNCR1-2
si-SLNCR1-NC
si-SLNCR1-1
si-SLNCR1-2
75
vimentin
A375
A875
si-SLNCR1-NC
si-SLNCR1-1
si-SLNCR1-2
si-SLNCR1-NC
si-SLNCR1-1
si-SLNCR1-2
60
GAPDH
A375
A875
si-SLNCR1-NC
si-SLNCR1-1
si-SLNCR1-2
si-SLNCR1-NC
si-SLNCR1-1
si-SLNCR1-2

Figure 5C

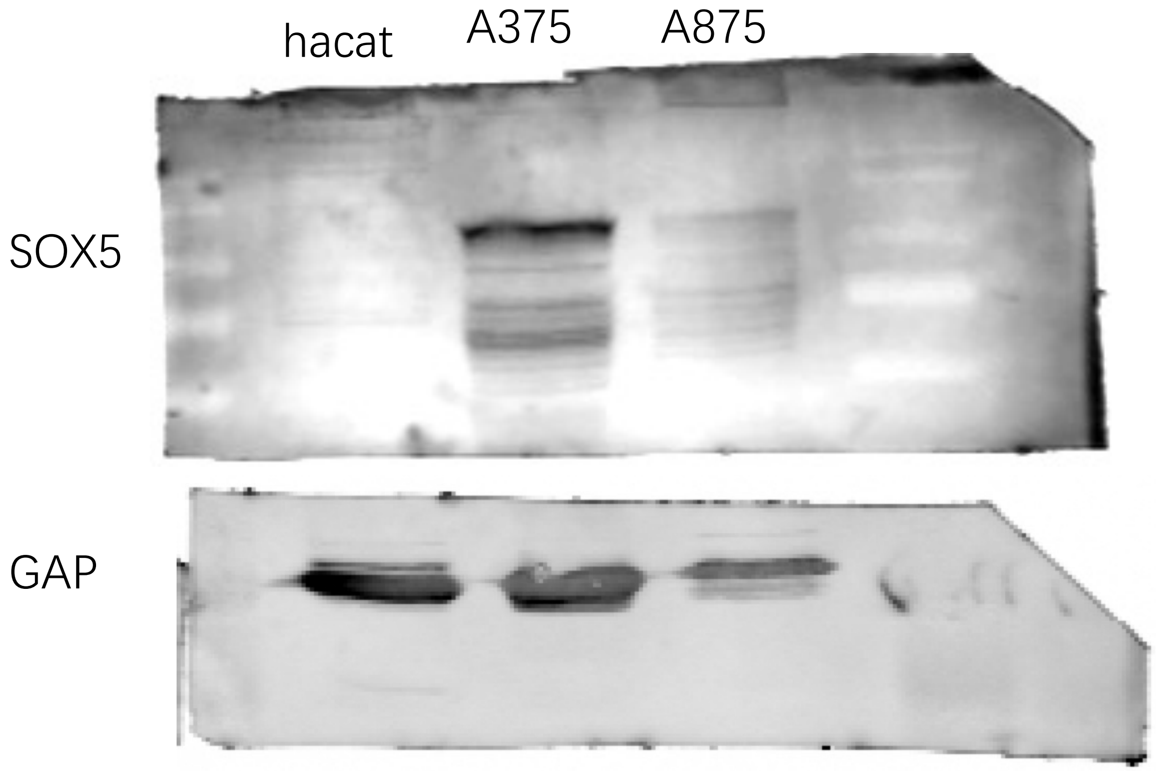

Figure 5I

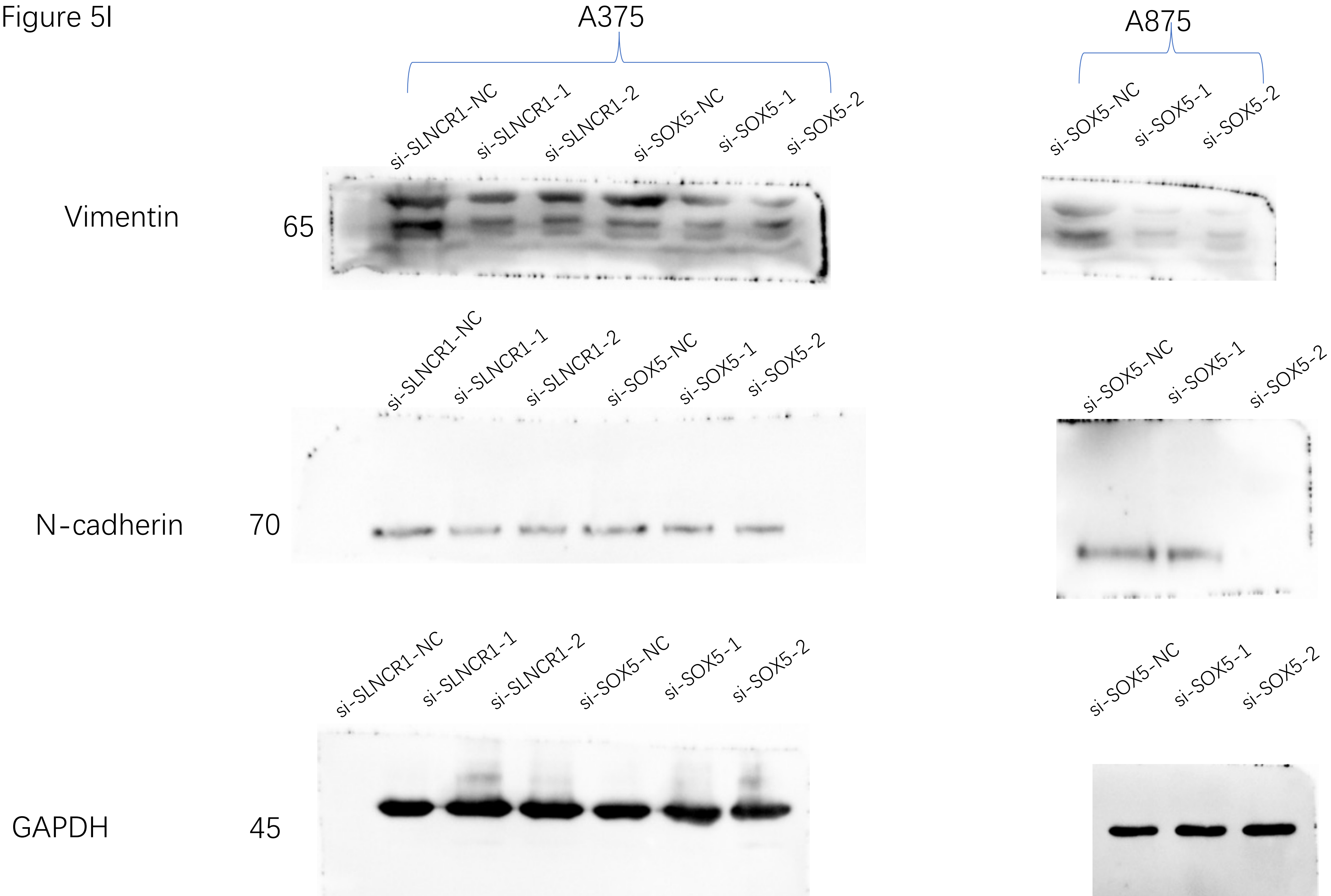

A375
A875
si-SLNCR1-NC
si-SLNCR1-1
si-SLNCR1-2
si-SOX5-NC
si-SOX5-1
si-SOX5-2
si-SOX5-NC
si-SOX5-1
si-SOX5-2
Vimentin
65
N-cadherin
70
GAPDH
45

Figure 6G

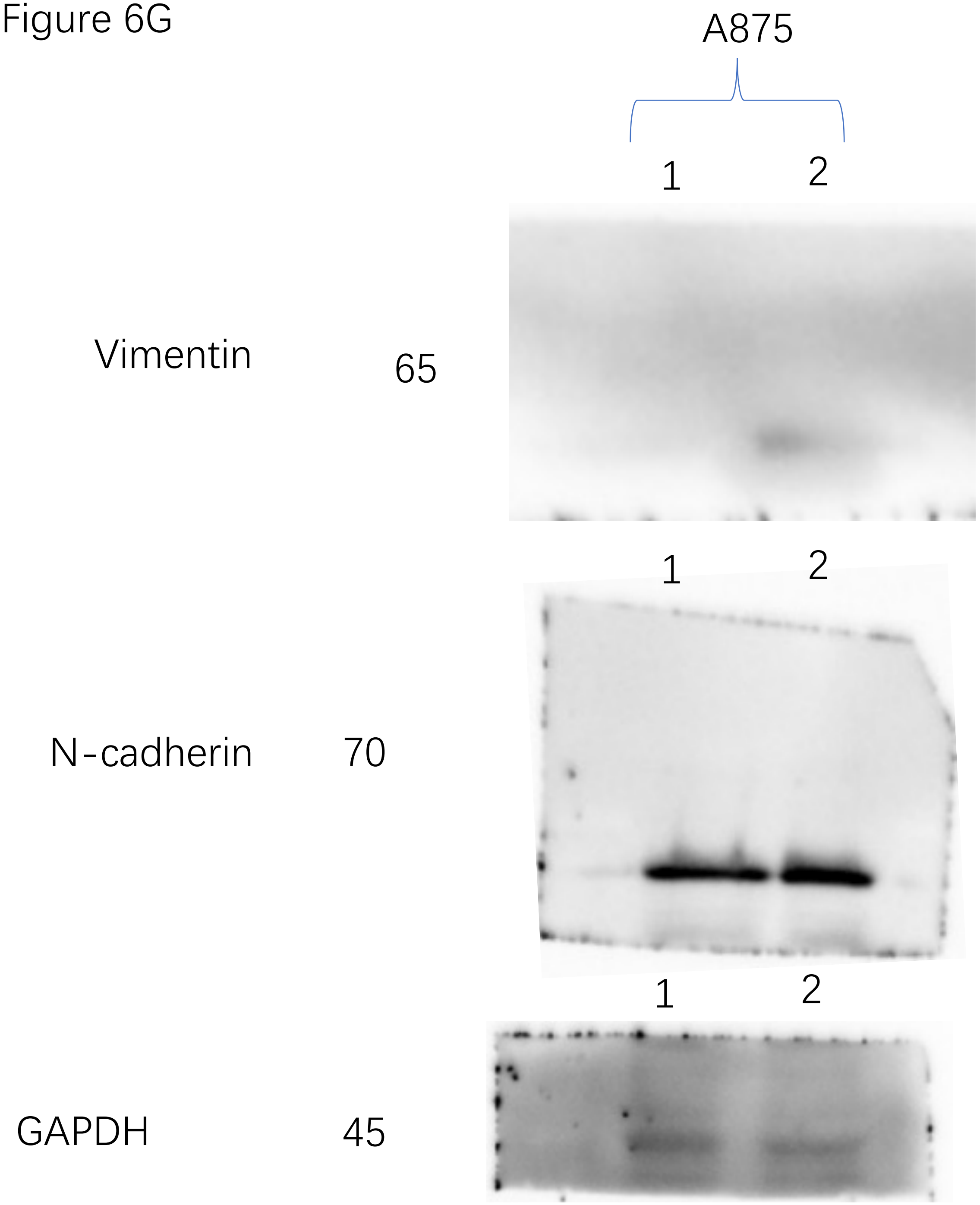


1：SLNCR1-sh-NC

2： SLNCR1-sh-SOX5-Over expressed
